# A review of chronic enterocolitis of rhesus macaques (*Macaca mulatta*) and potential as a naturally occurring model for post-infectious irritable bowel syndrome

**DOI:** 10.3389/fvets.2026.1759338

**Published:** 2026-04-01

**Authors:** Rebecca L. Bacon, Sara D. Lawhon, Stanton B. Gray, Carolyn L. Hodo

**Affiliations:** 1 Division of Laboratory Animal Resources, Duke University School of Medicine, Durham, NC, United States; 2 Department of Pathology, Duke University School of Medicine, Durham, NC, United States; 3 Texas A&M University College of Veterinary Medicine, College Station, TX, United States; 4 Southwest National Primate Research Center, Texas Biomedical Research Institute, San Antonio, TX, United States; 5 Michale E. Keeling Center for Comparative Medicine and Research (KCCMR), The University of Texas MD Anderson Cancer Center (MDACC), Bastrop, TX, United States

**Keywords:** *Campylobacter*, chronic enterocolitis, diarrhea, histology, inflammatory bowel disease, irritable bowel syndrome, *Macaca mulatta*, post-infectious irritable bowel

## Abstract

Chronic enterocolitis (CE) is a disease of significant concern in colony-housed rhesus macaques, leading to chronic diarrhea and severe weight loss necessitating euthanasia in up to 25% of adults. Despite over three decades of research into this syndrome, mitigating strategies have been met with variable success and chronic diarrhea remains an ongoing problem in research colonies. Some risk factors, clinicopathologic characteristics, and histopathologic characteristics have been investigated, but an underlying cause has yet to be determined, making identification of at-risk individuals and development of specific therapies difficult. There is some evidence for the syndrome occurring as a post-infectious sequela, particularly with respect to *Campylobacter* spp. infections, though associations with protozoal agents and other bacteria have been investigated as well. If causality is proven, the syndrome could be used as a naturally occurring model for post-infectious irritable bowel syndrome (PI-IBS) in humans, a syndrome which develops in over one-third of people following an episode of infectious gastroenteritis. Existing animal models fail to replicate PI-IBS in its entirety, preventing the development of effective therapies for this disruptive disease. Given the impact CE has on research colonies, particularly when macaques are in short supply for critical research, as well as the potential as a translational research model, further investigation into this syndrome is crucial. This review will aim to revisit the characterization of CE in rhesus macaques, provide a brief summary of existing animal models for PI-IBS, and discuss recent work on the suitability of CE as a model for the human disease.

## Introduction

Chronic enterocolitis (CE), also known as idiopathic chronic diarrhea (ICD), chronic idiopathic colitis (CIC), or chronic idiopathic diarrhea (CID), is considered the leading cause of morbidity and mortality in colony-housed rhesus macaques (*Macaca mulatta*), developing in up to 25% of adults and resulting in chronic diarrhea which can lead to nutrient malabsorption, loss of epithelial barrier function, growth stunting in young animals, and death (1–4). The cause is unknown and treatment, focused on rehydration and symptom management, is typically unrewarding, with animals ultimately requiring welfare euthanasia (4, 5). The syndrome has been studied for over three decades, but identification of the initiating factors and causes for ongoing disease has so far proved elusive. Numerous organisms have been implicated as initiators of disease, dietary intolerances have been investigated, and numerous risk factors explored (3, 5–8). However, predicting which animals are predisposed to developing the disease and subsequent management of the syndrome remains difficult with large knowledge gaps regarding the pathophysiology. This review will aim to discuss what is known about CE in rhesus macaques, particularly with respect to recent advances in the field, and address ongoing knowledge gaps and potential avenues for investigation. Additionally, there have been many investigations into the suitability of CE as a naturally occurring model for a variety of human chronic intestinal diseases, particularly post-infectious irritable bowel syndrome (PI-IBS). As such, these investigations will be discussed and the limitations of existing animal models for PI-IBS will also be briefly reviewed. Identifying a holistic, natural model of PI-IBS would be a major leap forward in our ability to investigate components of PI-IBS in humans. A more complete understanding of the diagnosis and management of CE would also allow us to improve the health of rhesus macaque colonies, a critical research resource.

## Clinical description and investigated risk factors

CE in rhesus macaques presents with intermittent or continuous, non-bloody diarrhea, negative results on infectious disease tests, and is generally refractory to treatment. Symptoms are usually accompanied by dehydration and weight loss (3, 9–11). The syndrome is typically diagnosed in animals less than 5-years-old, although it has been described in older animals. Some publications describe a male predilection, though specific case definitions utilized in studies vary widely, somewhat complicating consistent reporting of the syndrome (5, 7, 12–14). Critically, CE as a cause of an animal’s diarrhea must be differentiated from other potential causes of intestinal distress in via review of the clinical and husbandry history of the animal, fecal culture, and utilization of clinicopathologic and histopathologic data where appropriate. Johnson et al. provided a complete review of intestinal diseases of rhesus macaques in 2022, including common causes of diarrhea such as the bacterial agents *Campylobacter* spp.*, Shigella* sp., and *Yersinia* spp., parasitic agents including *Cryptosporidia* and *Trichuris* sp., and less commonly viral agents like cytomegalovirus or adenovirus. Non-infectious causes of diarrhea are less common but should be ruled out, particularly in chronic diarrhea cases, and include intestinal amyloidosis, cicatrizing ulcerative colitis, and colonic carcinoma. Some publications suggest CE may be a post-infectious syndrome, similar to PI-IBS in humans (2, 14–17). Reports of cicatrizing ulcerative colitis are rare, but it is also hypothesized as a post-infectious syndrome (3).

Numerous social and physical factors can increase the likelihood of an animal developing CE including diarrhea prior to weaning, low body weight at the time of weaning, movement from indoor to outdoor housing during the fall, hand-rearing, and being male (5, 7, 12). One study also found that animals that were of more anxious or gentle temperaments, those who had repeated housing relocations, and particularly those who fell into both categories were more likely to develop chronic diarrhea (13). Another found low ranking animals, those that spent more time separated from their group, and animals classified as being low in gregariousness were also predisposed. Again, this study also found males to be affected more frequently than females. However, animals were included in this study even if they had already had episodes requiring hospitalization, potentially resulting in failure of formation of social attachments which may skew these behavioral findings (5).

All of these factors have in common a presumed increase in individual stress and anxiety of an animal. In support of stress as a risk factor, in addition to behavioral characterizations, Howell et al. measured plasma cortisol concentrations and performed adrenocorticotropic hormone (ACTH) stimulation tests on affected animals in comparison to unaffected animals to assess response to stress and whether affected animals had a functional hypothalamic–pituitary adrenal (HPA) axis (5). While humans with inflammatory bowel disease and stress related IBS have shown increased cortisol levels and influence of daily stress on symptoms, in this study, CE animals had lower plasma cortisol compared to normal animals (18–21). There was an insufficient sample size to determine differences between ACTH groups, but CE animals tended to have lower baselines and higher ACTH stimulation levels. This may suggest down-regulation of the HPA axis, but there was also some indication that cortisol levels were linked with dominance rank which could account partially for the differences appreciated between rhesus macaques with CE and humans with inflammatory bowel disease (IBD) and IBS (5). Similarly, PI-IBS is reported to more frequently affect females than males, which may be considered opposite to what we see in rhesus macaques. However, a study in humans demonstrated that female predisposition for PI-IBS does not exist if psychosocial factors are controlled for in analysis, so it seems reasonable to suggest a similar explanation for the male predisposition in rhesus macaques with CE, but these investigations have not been performed (22).

Laing et al. attempted to more fully characterize the pathophysiology of CE and delineate parasitic, protozoal, or bacterial organisms that affected animals that developed the disease. The inclusion criteria for this study were animals that had more than 45 days of diarrhea and/or more than 3 hospitalizations for diarrhea over a period of 6 months, negative enteric pathogen screening in the prior 6 months, and no evidence of an infectious component to the diarrhea at the time of necropsy. Regarding infectious organisms, this study found that *C. coli, C. lari,* and trichomonads were isolated in high numbers from animals that progressed to have CE: 40% had *Campylobacter* spp. and 26% had trichomonads, while only 9% of control animals had *Campylobacter* spp. (in low numbers) at the time of necropsy, and 0% had trichomonads. However, the study did not specify whether positive cultures in animals were obtained before or after they were diagnosed with CE, somewhat complicating interpretation of the results. The authors also described a “blue brush border” lining the lumen of the large intestine, which is comprised of *Helicobacter macacae* and determined thatw a lack of these epithelial adherent organisms was consistent with dysbiosis in CE-affected animals (2, 3). While this study suggests a link between *Campylobacter* infection and CE, as a retrospective study, it does not establish a causal relationship or discriminate between animals that had a positive *Campylobacter* culture during an initial episode of diarrhea versus those that were culture positive during any episode of diarrhea throughout the disease process. Our recent study investigating *Campylobacter* sp. prevalence in a colony of rhesus macaques found a trend of increased *Campylobacter coli* or *jejuni* load in the feces, measured by qPCR, in animals with CE and in animals that went on to develop CE following sampling, but sample numbers were insufficient to assess significance. This study also reported certain multi-locus sequence types (MLST) of *C. coli* found only in animals with intestinal disease and some found only in healthy animals (15). These results support the Laing et al. hypothesis of *Campylobacter* involvement in the development of CE, but more investigations are needed (2, 15).

## Treatment

Historical treatments have included empiric treatment with fenbendazole, doxycycline, metronidazole, enrofloxacin, bismuth subsalicylate, and tylosin (5, 23). However, as widely accepted in human medicine, and proven specifically in rhesus macaques, antibiotic therapy can significantly alter the gut microbiome (24). These treatments are now most often used when the involvement of a specific pathogenic organism is strongly suspected or confirmed. Repeat antibiotic exposures are also known to shape the microbiome and result in permanent derangements with the potential to perpetuate intestinal inflammation (25). Regardless, tylosin particularly has received attention due to its use in canine chronic enteropathies, demonstrated success in the treatment of a colitis model in rats, and antimicrobial activity against various enteric organisms such as *Clostridium* spp., *Helicobacter* spp., and *Campylobacter* spp. (26–29). A study by Blackwood et al. in 2008 demonstrated improved diarrhea and colonic lesions following 10 days of parenteral tylosin therapy, and animals which received therapy for up to 6-weeks had normal fecal scores for the duration of the treatment period. However, nearly 40% of these animals experienced a relapse in symptoms within 30 days of cessation of tylosin therapy (23). It is also worth noting, tylosin therapy has been shown to result in dysbiosis, specifically a decrease in gut microbial diversity, even following one dose in both pigs and dogs, so it’s use should be considered carefully (30, 31). More recently, oral fecal bacterial transfer, dietary coconut, exposure to the helminth *Trichuris trichiura*, and the oral prebiotic therapy inulin, have shown some promising results, though these require further investigation (32–35). In 2018, a small study examined the efficacy of a 14-day combination treatment using vancomycin, neomycin, and fluconazole which demonstrated maintained improvement in stool consistency following cessation of treatment. Despite the success, the authors of this study did note the need for a larger sample size to confirm findings, as well as acknowledging this may not represent an ideal colony-wide treatment plan but may be best used in certain high-value cases (36).

There has been some preliminary work to develop a *Campylobacter* vaccine, both to the benefit of rhesus macaques and humans, as there is not current *Campylobacter* vaccine available for human use and a lack of strong experimental models to test them, despite the large number of *Campylobacter*-associated enteritis cases across the world every year and the long term effects it can have (14, 17, 37). One group tested a hydrogen peroxide inactivated *Campylobacter coli* in rhesus macaques. The vaccine induced an antibody response to bacterial flagellin, a highly conserved gene between circulating strains of *C. coli*. Vaccination resulted in significantly reduced diarrhea incidence, with an incidence rate of 9.8% in the unvaccinated group and only 1.96% in the vaccinated group, resulting in an estimated vaccine efficacy of 83%. Diarrhea history of animals prior to vaccination was not delineated in this study, though animals that developed diarrhea within 2 weeks of vaccination were subsequently excluded as that time was considered insufficient for development of an immune response (14). These results support *C. coli* as a causative agent of disease rather than simply a commensal or bystander agent, particularly as the rate of *C. jejuni* associated diarrhea in this colony is reportedly quite low. With *C. jejuni* as the primary *Campylobacter* pathogen in most countries, these results may be of minimal utility for the development of a human vaccine, though in a pilot study of a similarly designed *C. jejuni* vaccine, some cross-protection between both species was hypothesized. While vaccination for *C. coli* was protective against diarrhea in general, additional studies and time are required to determine if early vaccination for *Campylobacter* would be protective specifically for CE, which would be a major step in confirming the bacteria’s role in the development of this syndrome.

Overall, no treatment has proven universally effective or suitable for widespread use in rhesus macaque colonies. Best treatment practices remain a process of elimination based on individual responses, likely speaking to the multifactorial nature of this syndrome. Long term studies also tend to be hampered by the needs of individual colonies, with animals exiting colonies for research purposes at irregular intervals, preventing long-term follow-up. Further understanding of the initiating factors of the disease and pathogenesis would be greatly beneficial in identifying more widely applicable, successful treatment strategies.

## Gross lesions, histology, and clinicopathology

**Figure 1:**
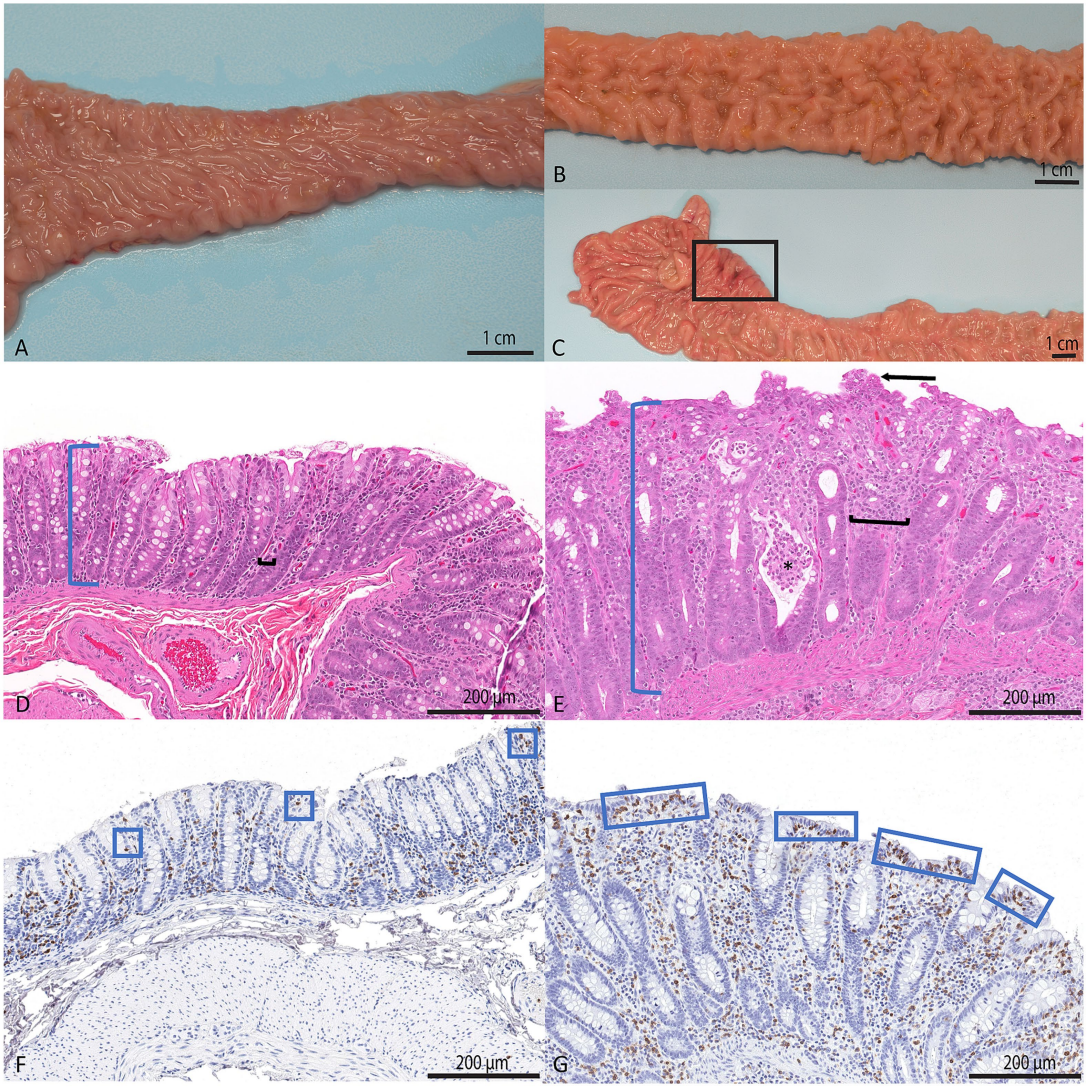
Classic gross and histologic features of rhesus macaques with and without CE. **(A–C)** Demonstrate the range of gross appearances of the colon **(A,B)** and ileocecocolic valvular region **(C)**. The colon in **(A)** is normal with shiny, healthy mucosa grossly, though this animal demonstrated histologic evidence of CE. The colon in **(B)** is thick and corrugated. The mucosa is rough and dull. The mucosa in **(C)** is similarly thickened and variably reddened with multifocal erosions (black box). **(D)** is a representative histologic section of normal colon. The blue bracket demonstrates the thickness of the mucosa and the black bracket demonstrates the colonic crypts separated by few resident lymphocytes. Hematoxylin and eosin, 20x. **(E)** is a representative histologic section of colon from an animal severely affected by CE. Classic features include proliferative mucosa (blue bracket), increased mononuclear cells between crypts (black bracket), crypt abscesses (asterisk), and irregularity and tufting of the luminal epithelium (arrow). Hematoxylin and eosin, 20x. **(F)** demonstrates normal quantities of intraepithelial lymphocytes (blue boxes) while **(G)** demonstrates significant increases in intraepithelial lymphocytes. Immunohistochemistry, CD3, 20x.

Gross lesions in animals with CE classically include a colon distended by watery, non-bloody material; mildly thickened, though generally not ulcerated, colonic mucosa; and enlarged colonic lymph nodes (2, 3). One report did note a difference in findings between animals that succumb to the disease as young adults and those that succumb as older adults over 10 years of age. The younger animals had thin, translucent, and inflamed small and large intestines with enlarged mesenteric lymph nodes, while the older animals had more thickened small intestine, and the large intestine was thick with apparent fibrosis and tended to be less obviously inflamed (5). Histologically, these animals have a lymphoplasmacytic colitis, with some reports discussing involvement of the small intestine, similar to microscopic colitis in humans and diarrhea predominant IBS, though others suggest the disease is focused primarily on the proximal colon (4, 16, 38–40). Other histologic findings include crypt abscesses, mucosal neutrophils, mucosal hypertrophy, loss of goblet cells, increased epithelial lymphocytes, epithelial attenuation or tufting, absence of epithelium-adherent bacteria, and increased trichomonad-like parasites, particularly extending into colonic crypts (2, 3). Diagnostic thresholds for the leukocyte populations, including intraepithelial lymphocytes, have been suggested in order to improve consistency in diagnostics and in reporting across studies (16). Laing et al. described histologic and immunologic changes in a cohort of rhesus macaques with CE. The affected animals in their cohort were primarily outdoor-housed macaques under 5 years of age. Using semiquantitative methods, they found enteroendocrine and enterochromaffin cells to be increased in density in the mid colon, but not the rectum, CD3 + intraepithelial lymphocytes were increased in the colon and small intestine, and Th2 cells were decreased in the colon, but there was no difference in CD4 + FoxP3 + cells. Our group corroborated increases in CD3 + intraepithelial lymphocytes in a separate study, but we found no difference in enteroendocrine and enterochromaffin cells between affected and unaffected animals (16). The method of assessment of these two cell populations differed between the two studies which may play a part in the varying results. Additionally, while the Laing study included only rhesus macaques under 5 years of age, our study included animals across all age groups. Despite the differences, both studies did identify lesions in the small and large intestine and agree on the increases in intraepithelial lymphocytes, a finding which in humans has been associated with previous infectious insults (41). Other studies have also shown alterations in the local and systemic immune response in chronic intestinal disease, with increases in activated T lymphocytes in gut-associated lymphoid tissue and increases in expression of IL-1α, IL-1β, IL-3, TNF-alpha, IL-6, and IL-8 (6, 42). Representative classic gross and histologic images of CE, including immunohistochemical characterization of intraepithelial T lymphocyte populations, are available in Figure 1.

Regarding clinicopathologic data, in addition to the cortisol and ACTH findings discussed above, abnormalities include elevated basophils, triglycerides, and urea nitrogen; and low sodium, potassium, and chloride (5). One study in rhesus macaques and pig-tailed macaques (*Macaca nemestrina*) looked at cobalamin levels, as low cobalamin is a commonly seen change in humans with Crohn’s disease or IBD and dogs with IBD or lymphoma, and can be associated with a poor outcome in those diseases. That study included animals of both species at least 3-years-old that had either persistent diarrhea for at least 6 months despite clearance of an infectious cause, intermittent diarrhea of at least 3 months with no infectious cause, or confirmed CE via endoscopic intestinal biopsy, and found decreased cobalamin and increased platelets in the pig-tailed macaques (*Macaca nemestrina*) but not in rhesus macaques with CE (10). As such, clinicopathologic abnormalities demonstrated to date appear directly related to the ongoing fluid loss secondary to chronic diarrhea and have not revealed predictive patterns for diagnosis or response to treatment.

## Infectious agents of interest

Over three decades of research into CE, numerous organisms have been implicated as initiators of disease, including *Campylobacter jejuni, Shigella flexneri, Yersinia enterocolitica, Strongyloides fulleborni,* simian type D retrovirus, and adenovirus, though none have as yet been definitively linked to CE development (5–8). Although, at the time of diagnosis, these animals tend to be negative for infectious organisms using standard culture techniques. One study attempted to determine if there was an underlying viral cause by looking at the enteric virome of rhesus macaques with CE, spurred by the notion that adenoviruses are an important cause of acute diarrhea in rhesus macaques, and simian immunodeficiency virus (SIV) infected macaques, another population prone to chronic intestinal disease, have shown higher enteric viral diversity (43, 44). They also suggested the lymphoplasmacytic inflammation could be indicative of a previous viral infection, but likened CE more to ulcerative colitis than to PI-IBS. This study suggested picornaviruses were weakly associated with CE and parvoviruses were weakly associated with a healthy status, but more recent studies have disproven this and shown no difference between the virome of CE and healthy animals. However, as in the SIV animals, the overall viral burden tends to be higher in animals with chronic diarrhea, which is indicative of an overall dysbiosis in CE animals (45, 46).

As referenced above, *Campylobacter* spp. are commonly implicated in the development and or maintenance of CE in rhesus macaques, though whether *Campylobacter* is an initiator of disease or an opportunistic bystander in CE in macaques remains to be determined. As such, *Campylobacter* spp. have received significant attention when investigating diarrhea in rhesus macaques. Macaques experience an acute campylobacteriosis syndrome similar to humans, particularly with infection by *C. jejuni*. Macaques can develop diarrhea which is sometimes bloody, inappetence, weight loss, and lethargy. Histology of acute infections displays an acute neutrophilic and lymphocytic colitis with cryptitis (3, 47). One study in pig-tailed macaques also showed that similar to human infections, *C. jejuni* infection produced elevations in IgA, IgG, and IgM which were protective to future exposure from the same *C. jejuni* strain, but a future study by the same group showed protection did not extend to different strains, with multiple reinfection events with different strains in a group of infants in a *Campylobacter* endemic environment (47, 48). Other similarities between macaque and human *Campylobacter* infections are that infection also results in intestinal dysbiosis, there are symptomatic and asymptomatic carriers, and infants appear to experience a higher disease incidence and severity than adults (14, 49, 50). In some colonies, up to 80% of outdoor housed rhesus macaques are colonized with *Campylobacter* species by 1 month of age and over 69% of adults and juveniles will remain asymptomatic carriers of *C. coli* and/or *C. jejuni,* a higher proportion than is seen in humans. Up to 25% of infected infants develop *Campylobacter*-associated diarrhea and half of these animals go on to develop CE (14, 49). Additionally, there can be multiple *Campylobacter* strains circulating in a population, even within the same individual. One study in pig-tailed macaques using a combination of polyacrylamide gel electrophoresis, serotyping, and DNA hybridization found 10 serotypes in a group of 69 isolates, with infant macaques harboring an average of 8 different *Campylobacter* strains (48). Given the high prevalence even in healthy animals, there is some debate of the role of *C. coli* as a primary pathogen. Using metatranscriptomics, Westreich et al. showed that animals with chronic diarrhea harbored *Campylobacter* with higher virulent gene expression, including genes for adhesion, motility, and chemotaxis, and *Campylobacter* was more closely associated with the mucosa in these animals than in controls. They also found the amount of pVir plasmid, a plasmid which carries genes homologous to type four secretion system genes in other intestinal pathogens and appears to be involved in adhesion and invasion, to be four times higher in samples from animals with CE compared to those without. While there is no doubt that interplay with the host immune system drives selection of these more virulent phenotypes, these results also suggest potential strain level differences involved in which animals develop CE and which do not (51). Arguably the most important virulence factor for the development of acute colitis is cytolethal distending toxin (Cdt) which causes epithelial cell cycle arrest, cell distension, cell swelling, and apoptosis, ultimately contributing to the release of a large amount of the proinflammatory cytokine interleukin-8 (IL-8) from epithelial cells and epithelial barrier disruptions. *Campylobacter* can also express a Cas9 nuclease which contributes to stimulating apoptosis and IL-8 release. This abundant IL-8 release results in the recruitment of large numbers of neutrophils resulting in a large amount of the tissue damage associated with acute campylobacteriosis (52).

Similar to human medicine, genetic analysis of *Campylobacter* isolates from rhesus macaques is just starting to come to the forefront of investigations. Whole genome sequencing reports of *Campylobacter* isolates from rhesus macaques are sparse, but this is a gap that needs to be filled if we are to truly understand the contributions of *Campylobacter* spp. to chronic disease in this species and determine the applicability of the rhesus macaque CE syndrome as a model for PI-IBS. One genome announcement from an isolate from a rhesus macaque at a research center in Arkansas, USA found a *C. coli* which had hybridized content from *C. jejuni,* as well as a plasmid which contained two resistance genes, *tet(O)* and *oxa-61,* for tetracyclines and *β*-lactamases, respectively (53). These hybrid strains may grow in importance as hybridization appears to increase the ability of strains to cross between host species and increase viability in an intestinal environment, as shown by a separate group investigating hybrid strains of *C. coli* isolated from poultry (54). Another study, just from early 2023, described partial genome sequencing of 13 isolates from wild rhesus macaques with diarrhea in Nepal, from a larger group of 67 diarrheic samples, 96% of which were PCR positive for *Campylobacter* species. The sequence results of the PCR products yielded *candidatus C. infans* and *C. helviticus* (55). While this group did not eliminate other cause of diarrhea in the population, both of these species have been reported to cause diarrhea in humans and this supports the notion mentioned previously that unusual or novel species of *Campylobacter* should potentially be investigated in rhesus macaques alongside the more typical species of *C. coli* and *C. jejuni.*

## Microbiome

Aside from histologic and immunologic characteristics, rhesus macaques also display enteric microbiome commonalities with chronic inflammatory intestinal diseases in humans like IBD and PI-IBS, which often result in decreased microbiome diversity. Animals with chronic diarrhea demonstrate dysbiosis when compared to unaffected animals, with one study showing decreased levels of *Lactobaciillus* spp. but increased levels of mucin-degrading bacteria and pathogenic bacteria such as *Clostrioides difficile* (2, 25). This dysbiosis presumably contributes to maintenance of disease and inflammation. A recently emerging area of interest within dysbiosis evaluations is specific attention to the antibiotic resistome, or the collection of antibiotic resistance genes present in the gastrointestinal tract. There are some suggestions in rhesus macaques and humans with chronic intestinal disease that the resistome may contribute to driving inflammation and therapeutic responses (25, 56, 57). As in PI-IBS however, the role of the resistome can be difficult to separate from the overall effects of dysbiosis as individuals with more severe disease are more likely to receive antibiotics as an adjunct therapeutic, further affecting microbial communities and frequency of resistance genes (58). When focusing on initiating factors of CE, microbiome studies also often support *Campylobacter*’s potential role in CE (1, 59–61). In healthy animals, Rhoades et al. showed the rhesus macaque microbiome is similar to the microbiomes of humans in developing or non-westernized countries, a finding also corroborated by a more recent evaluation of the rhesus macaque gut microbiome in health and chronic diarrhea (1, 25). Rhoades et al. showed rhesus macaques to containin abundant *Prevotella, Ruminococcus,* and *Treponema* species and lacking *Bacteroides,* though the density of *Treponema* species has been described as somewhat unique to rhesus macaques in other studies (1, 59). This group also followed outdoor-housed infant rhesus macaques throughout the first 8 months of their life, and demonstrated that animals that developed diarrhea had unique *Prevotella* strains prior to disease, those that developed disease had a larger *Campylobacter* burden, and some *Campylobacter* species are only found in animals that had diarrhea. However, in this study, no samples were compared after diarrhea but before treatment, so the imbalances in *Campylobacter* could potentially be due to treatment related alterations in the microbiome. An earlier study on the rhesus macaque microbiome by McKenna et al. found increases in *Campylobacter* in animals with colitis, with 50% of animals with colitis showing increases compared to none of their seven control animals, and in this study, these changes were specifically not related to antimicrobial administration. Crucially, only two of the animals showing *Campylobacter* increases on microbiome assays were positive for *Campylobacter* sp. using culture methods, signifying a need for molecular diagnostics as part of standard diagnostic protocols (59). A secondary outcome of the Rhoades et al. study showed that the age of the animal was a much more significant factor in microbiome differences than from which institution they came, suggesting that studies of bacterial populations in rhesus macaques in one institution may be able to be extrapolated to other institutions. The diarrhea cases in this group were diagnosed primarily as *C. coli* associated, with fewer episodes associated with *C. lari, Shigella,* or *C. coli* and *Shigella* together, though in 17% of cases no infectious organism was identified. The 8-month-old macaques that had experienced diarrhea and treatment had higher *Campylobacter* load and the asymptomatic group had higher *Helicobacter macacae* load, the same bacteria described as a crucial component of the blue brush border by Laing et al., supporting a hypothesis of potential niche competition between these two bacterial species (1–3). The McKenna et al. group demonstrated these changes in chronic colitis as similar regardless of whether an animal had SIV related colitis or not, suggesting microbiome changes are more referable to the presence of inflammation rather than the cause of the inflammation, but again, did show associations with the presence of *Campylobacter* species and chronic colitis. Interestingly they also found one animal with chronic diarrhea which had been negative for infectious agents on standard screening tests to be carrying *C. fetus* and *C. hyointestinalis* supporting the importance of investigating less frequently encountered *Campylobacter* species (59).

A more recent study comparing fecal microbiota in healthy free-ranging rhesus macaques to that of outdoor housed captive rhesus macaques with and without CE found few differences between the captive populations except for an abundance of Proteobacteria in animals with CE, but found significant differences between captive and free-ranging animals including large populations of Christensenellaceae in the free-ranging animals, a family also associated with healthy status in another study, and *Helicobacter* sp., which are associated with healthy microbiota profiles in humans (36, 62). This study excluded animals from the study pool if antibiotics had been used in the months prior to collection, so alterations due to antibiotics were not expected, a known limitation of the previous studies. Also, in contrast to the Mckenna study, this study did not separate males and females for analysis, which could mask some differences given the potential male predisposition for CE (59, 62). They found a higher proportion of *Bacteroides* in the free-ranging population than in the captive populations and as compared to other publications in captive populations. Their findings support the protective role of *Helicobacter macacae* in GI homeostasis. This study also supported the niche displacement theory, where *H. macacae* is lost and displaced by *Campylobacter* sp. in diarrheic animals, explaining the inverse relationship of these two bacteria in healthy and symptomatic animals. Overall, this study suggests that housing strategy has a greater influence on the gut microbiome than health status, potentially explained by differences in diet and stress level. The free-ranging macaques, while fed monkey chow, had access to a greater abundance of roughage than corralled macaques, and stress levels likely differed between the free-ranging and corralled populations because of differences in human and social interaction. This suggests, that while bacterial and host factors are important, modulating external factors may be a more rewarding treatment modality than specific treatment of individuals. Recent efforts at diet modulation, particularly with respect to limiting lactose content and increasing fiber content, has shown some success at mitigation of chronic diarrhea in rhesus macaques (63, 64).

## Review of PI-IBS

PI-IBS is a complicated, multifactorial disease in humans which can occur after any episode of infectious gastroenteritis, though *Campylobacter, Escherichia coli, Salmonella,* and *Shigella* species are the most commonly involved organisms. The syndrome was first described in 1962 but did not receive much research attention until the 1990s (65, 66). Some studies show a higher rate of PI-IBS following parasitic or protozoal enteritis than is associated with bacterial agents, but the symptoms tend not to be as severe or as long lasting (67–69). PI-IBS results in chronic, low-grade intestinal inflammation, mucosal dysfunction and increased permeability, and changes to the intestinal microbiota, particularly a decrease in microbial diversity. More specifically, there tend to be increased populations of Proteobacteria, the phylum containing the genera *Pseudomonas, Campylobacter,* and *Klebsiella*, and Firmicutes, containing *Bacillus, Clostridium, Streptococcus, Enterococcus, Staphylococcus,* and *Lactobacillus,* but reduced populations of Actinobacteria, containing *Streptomyces* and *Bifidobacterium*; and of Bacteroides, containing *Bacteroides* and *Prevotella* (66, 67, 70–72). The severity and duration of the initial infectious episode, as well as host factors such as the female sex, pre-existing anxiety or depression, and young age play a role in an individual’s risk for developing PI-IBS (58, 67, 68, 73). One study that demonstrated if psychologic variables and sex were included in multivariate analysis, the female predisposition was not a significant independent risk factor, but most studies still include a female predisposition in their descriptions of the disease (22). Some studies have also reported antibiotic use as a risk factor, particularly regarding the development of PI-IBS in children, but other studies suggest it is difficult to separate antibiotic use from severity as disease as more severe cases receive empiric antibiotic treatment, so this may be a false assumption (58). Certain genetic polymorphisms have also been reported in humans with PI-IBS, particularly in genes for TLR9, IL6, and CDH1, which are involved in pathogen recognition, immune response, and the integrity of the intestinal epithelium, respectively (67). A recent meta-analysis by Svendsen et al. evaluated whether or not the specific pathogen causing the original episode of acute gastroenteritis played a major role in the development of PI-IBS (58). This study looked at *Campylobacter* spp., *Clostridium difficile, E. coli,* Shiga-toxigenic *E. coli, Salmonella* spp., *Shigella* spp., *Yersinia enterocolitica,* rotavirus, norovirus, *Entamoeba histolytica*, *Giardia intestinalis,* and *Cryptosporidium* spp. They reported 17% of people developed PI-IBS after any episode of acute gastroenteritis and concluded that the different bacteria species did not have inherent differences in the risk of causing PI-IBS (58). However, this study only looked at differences at the species level and there was an overall low number of papers included for each infectious organism with many studies lacking unaffected control groups. Strain level differences are known to be risk factors for development of other post-infectious sequelae following *Campylobacter*-associated diarrhea and should not be dismissed as potential independent risk factors for PI-IBS. As an example, some strains of *Campylobacter* can express a lipooligosaccharide that acts as a ganglioside mimic, resulting in the molecular mimicry which induces Guillain-Barré syndrome (74). The antibodies that are produced against these ganglioside mimics ultimately cross-react with host gangliosides, resulting in axon degeneration and demyelination, particularly of motor neurons (75). Few studies have looked at similar possibilities regarding PI-IBS and *Campylobacter* specifically, but sub-species level differences have been identified that may predispose people to developing PI-IBS. These will be further explored in following sections.

Regarding the adaptive immune system, *Campylobacter* infection results in IgA, IgG, and IgM antibody production, but antibody levels decrease within several months and individuals are not generally protected against future infection by different serotypes, a feature which has been a major roadblock for vaccine development, and one which can hinder definitively associating an episode of acute campylobacteriosis with the development of PI-IBS (76–78). Cell-mediated immunity appears to be a crucial component of resolving *Campylobacter* infection but some have proposed that this response is also critical to the long lasting effects *Campylobacter* infection can have in humans, particularly resulting in increased numbers of γδ CD8 + T cells which are associated with cytotoxicity and autoimmunity (52, 78–81).

PI-IBS is characterized by some specific physical changes which set it apart from IBS and other chronic inflammatory diseases of the intestine. The microscopic lesions of PI-IBS include colonic mucosal hyperplasia along with lymphocytic and eosinophilic infiltrates, which may indicate some cross-over with low-grade inflammatory bowel disease (82, 83). The lymphocyte populations are comprised of increased proportions of CD3 + T cells and CD4 + T cells, particularly CD45 + memory T cells and double positive CD4 + CD8 + cytotoxic T cells, and decreased proportions of CD19 + B lymphocytes compared to samples from healthy controls (39, 83, 84). In addition to increases in the lymphocyte population in the lamina propria of the intestine, some reports also describe increases in mucosal mast cells as well as intraepithelial lymphocytes (IELs) (85). Mast cells produce substances like tryptase, histamine, and serotonin, which can activate enteric nerves. In IBS as a whole, the quantity of mast cells, particularly near enteric nerves in the lamina propria, correlates with the severity of visceral pain a patient experiences with IBS (86, 87). Mast cells represent intermediaries between other inflammatory cells and the enteric nervous system and can be activated by pathways typically associated with allergic reactions (e.g., the IgE pathway) or by numerous other pathways including activation of toll-like receptors (TLRs) (84). IELs are primarily T lymphocytes, though there are numerous subsets with complex phenotypes and varied functions. Classically, increases in IELs are associated with previous infectious insults, as well as with celiac disease (41).

More recently described microscopic changes in PI-IBS include increased intestinal permeability, increased enteroendocrine cells (EECs), and increased enterochromaffin cells (ECs). Increased intestinal permeability allows continued uptake of antigenic material, resulting in increased or ongoing inflammation and is classically measured in humans via oral administration of lactulose and mannitol with subsequent metabolite measurements in the urine, though other methods are utilized in experimental settings using tissue biopsies (88). Intestinal EECs are found only in the mucosa and generally represent only 1% of the epithelial cell population of the intestinal mucosa. In humans, EECs are found most frequently in the proximal intestinal tract, are lowest in frequency in the colon, and then the proportion increases again significantly in the rectum. EECs are identified on histology using immunohistochemistry for either synaptophysin or chromogranin A and there are three main types characterized by their respective secretory products: the enterochromaffin (EC) cell which produces serotonin (5-HT), the D cell which produces somatostatin, and the L cell which produces peptide YY (PYY), glucagon-like peptide-1 (GLP-1), GLP-2, glicentin, and oxyntomodulin. The ECs represent the largest portion of EECs, often comprising up to 70% of the large intestine EEC population. Regarding function of these secretory products, serotonin is involved in intestinal motility and secretion, visceral sensation, and appetite; somatostatin is inhibitory for digestive and exocrine function and stimulates colonic peristalsis; PYY suppresses appetite and inhibits peristalsis but stimulates enterocyte proliferation; GLP-1 delays gastric emptying and is involved in postprandial satiety; GLP-2 also promotes enterocyte proliferation; glicentin both promotes enterocyte proliferation and inhibits gastric emptying; and oxyntomodulin solely inhibits gastric emptying (89). EECs overall represent part of the enteric nervous system and within PI-IBS have been primarily considered to be involved in the hypermotility symptom of PI-IBS (38, 39, 85, 90, 91). This hypermotility appears to be driven by the increase in proportion of ECs (EC hyperplasia) resulting in increased serotonin secretion and availability in the intestine, though given their basic roles, the other EEC secretory products likely also play a role in hypermotility and mucosal hyperplasia even though they have not been specifically studied. Serotonin has been the focus of primary investigations given the potential role of serotonin blockers as potential effective therapeutics. As referenced above, the actions of serotonin are many, stimulating pancreatic and intestinal secretions, enterocyte secretion, peristalsis, and being involved in nociceptive pathways (89, 90). The enterochromaffin cell increase is proposed to result in changed serotonin signaling, resulting in hypermotility, hypersensitivity, and increased gut permeability (38, 39, 72, 92–95). Pre-existing natural elevations in EEC populations in an individual can also create an inherent increased risk for developing PI-IBS following an episode of acute infectious gastroenteritis (72).

Diagnosis of PI-IBS can represent a challenge, but recent guidelines characterize the condition as the onset of new, Rome-criteria-positive IBS symptoms occurring following an episode of acute gastroenteritis. The Rome criteria were developed by the Rome Foundation, a not-for-profit organization that is specifically devoted to the development of research and education on disorders of gut-brain interaction, previously known as functional gastrointestinal disorders. Briefly, the Rome IV criteria for diagnosing IBS are abdominal pain, occurring at least 1 day per week within the previous 3 months, associated with at least two of the following: relief with defecation, changes to stool frequency, or changes to the appearance of the stool. Further subtyping based on the predominance of the type of stool appearance and defecation habits are also possible (i.e., constipation predominant, diarrhea predominant, mixed, and unclassified) (66, 96). An individual must not have had pre-existing IBS symptoms to qualify as affected by PI-IBS and symptoms must be present for at least 6 months prior to diagnosis. Other diseases which should be considered during the diagnosis of PI-IBS are inflammatory bowel disease (IBD), with the subsets of Crohn’s disease and ulcerative colitis, celiac disease, microscopic colitis, and tropical sprue. Certain clinicopathologic tests can help in discrimination, with fecal calprotectin used in the diagnosis of IBD and microscopic colitis, serologic testing for celiac disease, and colonoscopy with histopathology if needed for any of these syndromes (66). Regarding differentiation of these diseases by histopathology, Crohn’s disease is characterized by non-caseating granulomas; ulcerative colitis by transmucosal chronic inflammation, basal plasmacytosis, crypt distortion and inflammation, and mucosal ulceration; celiac disease by intraepithelial lymphocytosis, chronic mucosal inflammation, and villous atrophy; and microscopic colitis by either increased intraepithelial lymphocytes without crypt distortion (lymphocytic colitis) or by a continuous subepithelial fibrous band underneath the epithelium (collagenous colitis), with the caveat that there can be overlap in the histologic appearance of these syndromes (97–99). Tropical sprue can look very similar to PI-IBS histologically and geographic residence of the infected individual is important in this diagnosis. Tropical sprue is most prevalent in the tropical areas of Puerto Rico, Haiti, Cuba, India, and Pakistan (100). The inflammation in PI-IBS should typically be lower than is seen in many of these diseases, however, and in contrast to inflammatory bowel disease, PI-IBS should not include neutrophils as a component of the inflammatory milieu (72).

Treatment modalities are selected on an individual basis, but often include dietary management with limitations of certain food categories, psychological therapy including pharmaceutical treatment, and symptom specific pharmaceutical treatment including antispasmodics, anti-diarrheals, probiotics, and serotonin receptor agonists (66). Overall, PI-IBS is a complex and challenging disease, with large infectious enteritis outbreaks providing the best opportunity for prospective research. The Rome Foundation working team identified research areas of need as mechanistic pathogen-specific subgroup studies, investigations into potential biomarkers for PI-IBS diagnosis and prevention, and dietary and pharmacologic clinical trials, all of which require a well-characterized, holistic animal model (66).

## Potential of CE as a model for PI-IBS

PI-IBS is a worldwide health problem, one for which significant knowledge gaps exist, including unknown strain specific contributions of *Campylobacter* sp. to disease development, and the lack of an animal model encompassing all aspects of the disease. Animal models that can be easily studied and manipulated are critical for investigating the disease pathogenesis and potential treatment modalities. The progress in the development of animal models for PI-IBS was summarized in 2011 by Qin et al. and reiterated without major adjustments by the Rome Foundation in 2019 (66, 101). Commonly used methods include infection of laboratory rodents with intestinal parasites or bacteria, or oral administration of chemical substances. Most of these models have significant shortfalls as reliable models for PI-IBS. Regarding chemically induced models, the majority appear to induce changes more consistent with ulcerative colitis and as the body can only respond to insults in specific ways, the utility of simply inducing inflammation is unclear. Though these chemical agents do tend to induce long-lasting changes, particularly with respect to visceral hypersensitivity and motility disturbances, they do not appear to truly affect the permeability of the intestine as occurs PI-IBS (101). In the opinion of the Qin group, the best model is the trinitrobenzene sulfonic acid model which induces a Th1 immune response in rats and mice following infusion of the product into the colon. This model mimics the visceral hypersensitivity, motility dysfunction, histopathologic changes, increases in permeability, and secretory changes, of PI-IBS, with these changes lasting for up to 17 weeks after administration. However, while this model does create a controlled, reproducible environment, it neglects the primary source of these changes in humans, the infectious component, and completely disregards the bacterial or parasitic factors, that may contribute to disease development. While the disease is multifactorial and understanding the minutiae of the intestinal immune response is critical, the contribution of specific pathogen factors cannot be ignored as these may represent easily controllable risk factors PI-IBS.

Regarding attempts to develop models using infectious agents, infection of mice with *Salmonella* sp. and *Citrobacter rodentium* induced short-term changes in serotonin levels and some inflammatory mediators, but the changes normalized rapidly (92, 102, 103). Numerous attempts have been made to use *Trichinella spiralis* infection in mice and rats, but significant changes, particularly long-lasting intestinal hypersensitivity changes were only demonstrated in the NIH/Swiss strain of mouse. Additionally, *Trichinella* sp. infections in humans are uncommon, with the CDC estimating only 10,000 human infections annually worldwide, again making the application of this model to human medicine questionable. Infections with *Giardia duodenalis, Cryptosporidium parvum,* and *Nippostrongylus brasiliensis* have also been tried, but parasitic infections tended to only cause histologic changes in the jejunum, whereas histologic changes in PI-IBS primarily occur in the large intestine (104–109).

An ideal model would use an agent that affects a large portion of the human population every year and is known to trigger PI-IBS, like *Campylobacter*, to more closely investigate the infectious agent characteristics involved in the development of the disease. Regarding recreation of PI-IBS with infection by *Campylobacter* spp. specifically, Pimentel et al. attempted to infect rats with *C. jejuni*. Even 3 months after clearance of the bacteria, a significant percentage of the rats displayed a change in stool consistency, bacterial overgrowth, lower body weight, and increased rectal and colonic intraepithelial lymphocytes (110). However, as described by Qin et al. and others, this model has not been proven to replicate the changes in motility, intestinal permeability, or visceral sensitivity as seen in PI-IBS (66, 101). Despite the limitations and further investigation needed into the *C. jejuni* rat model, it has been used to display the potential importance of cytolethal distending toxin (Cdt) in the development of PI-IBS-like symptoms. In 2015, Pimental et al. determined that antibodies to the CdtB subunit cross-reacted with an enteric neural protein vinculin which is located in the interstitial cells of Cajal and myenteric ganglia, both of which are required for normal intestinal motility. Vinculin is also a component of cell junctions and important for structural integrity of cells. The authors hypothesized that autoimmunity induced by molecular mimicry of antibodies to CdtB which then attack vinculin may be responsible for some of the characteristics of PI-IBS, similar to the ganglioside mimic responsible for Guillain-Barré syndrome (74, 111). This group subsequently proved the importance of CdtB in the pathogenesis of the disease by injecting rats with CdtB only, in the absence of an infectious organism, and they developed a similar PI-IBS phenotype and again, autoimmunity to vinculin (112). This work has resulted in development of a diagnostic assay for PI-IBS involving the detection of anti-CdtB and anti-vinculin antibodies which appear to be able to distinguish diarrhea-predominant PI-IBS from inflammatory bowel disease, but the tests are still being validated and do not appear to be widely used as yet (113–115). These tests could become a component or requirement for qualifying any future animal models as ideal for PI-IBS. The Rome Foundation working team appears to favor the *Trichinella* and trinitrobenzene sulfonic acid induced models, though arguably, these considerations do not give sufficient weight to the rat *C. jejuni* model (66). Regardless, with no model reproducing every characteristic of PI-IBS, there remains a need for an animal model which encompasses all aspects of PI-IBS without genetic manipulation and ideally, one in which disease is caused by a more commonly encountered human pathogen.

A naturally occurring non-human primate (NHP) model of PI-IBS which replicates all aspects of the disease would be ideal for study in conjunction with these rodent models, given the anatomical and genetic similarity of NHPs to humans and their potential role in drug development and approval processes (116). The syndrome of CE in rhesus macaques has the potential to fill this gap (Table 1). It has been studied for several decades but conflicting evidence remains and many aspects of the disease are just now being elucidated with the increased use of genomic tools. The syndrome has been compared to many chronic intestinal diseases in the past, but the primary considerations now are PI-IBS and ulcerative colitis. A major roadblock in categorizing CE as one of these diseases has been a lack of systemic histologic evaluations of affected animals, comparison to human biopsy findings, and consistent case definitions across studies. Most of the rhesus macaque studies mentioned above used different clinical criteria to classify an animal as having CE, and few corroborated their study groups with histology. One human study mentioned several different chronic intestinal diseases as sequelae to *Campylobacter* infection, so histologic confirmation, once the histologic characteristics are clearly defined across study populations, ideally should be a component of future investigations. While the histologic changes can overlap between chronic intestinal diseases, classically, PI-IBS includes colonic mucosal hyperplasia, CD4 + lymphocytic predominant and eosinophilic inflammation, often increases in mast cells and intraepithelial lymphocytes, and increased EECs and ECs. Neutrophilic infiltrates should generally not be a feature. Progress towards standardized histologic diagnosis of CE is being made, with one study on one captive population of rhesus macaques further characterizing the histologic cellular components of CE (2). While the authors reported colonic mucosal hyperplasia, increases in intraepithelial lymphocytes, and increases in EECs and ECs in a single portion of the colon, they did not find increases in CD4 + T lymphocyte cytokines and did not describe the remainder of the cell populations involved, which are important in validating the disease as a model for PI-IBS versus other chronic intestinal diseases. Our group developed proposed semi-quantitative guidelines for diagnosis based on observed quantities of leukocytes, intra-epithelial lymphocytes, and other distinctive histologic features such as crypt abscesses, foci of crypt distortion and inflammation. In our work, while many features were similar between CE and PI-IBS, we found neutrophils to often be a component of the inflammatory milieu, contrary to what is reported for PI-IBS (16). The Nancy Index has been used for similar syndromes in other primate species, including Western lowland gorillas (*Gorilla gorilla*) and Sulawesi crested macaques (*Macaca nigra*), and may be applicable in rhesus macaques, though to date, no data utilizing this index in rhesus macaques has been published (9, 117). Utilizing the proposed guidelines in conjunction with histologic data from other studies across a wider population of colony-housed rhesus macaques may lead to improve detection and classification of the disease, though these methods are typically used in post-mortem samples and validation on pre-mortem biopsy samples may provide more actionable information both from a diagnostic and research standpoint.

**Table 1:** Comparison of diagnostic features of post-infectious irritable bowel syndrome and chronic enterocolitis based on current available literature.

Diagnostic feature	PI-IBS	CE
Species affected	Human	Rhesus macaque
Chronic hypermotility/diarrhea	Yes	Yes
Alterations to intestinal microbiome	Yes	Yes
	Decreased diversity	Decreased diversity
	Increased *Pseudomonas, Campylobacter, Klebsiella*, *Bacillus, Clostridium, Streptococcus, Enterococcus, Staphylococcus, Lactobacillus*	Increased *Campylobacter*
	Reduced *Bacteroides, Prevotella, Streptomyces, Lactobacillus*	Reduced *Prevotella,* Christensenellaceae
	Reduced *Helicobacter* spp.	Reduced *Helicobacter macacae*
Psychosocial components	Stress	Hand rearing
	Anxiety	Low position in social hierarchy
	Depression	Anxious temperament
	Other psychiatric comorbidities	Captivity and/or repeated relocations
Histologic findings	Colonic mucosal hyperplasia	Colonic mucosal hyperplasia
	Crypt dilation and inflammation	Crypt dilation and inflammation
	Variable epithelial disruption	Variable epithelial disruption
	Increased CD3 + T lymphocytes	Increased CD3 + T lymphocytes
	Increased CD4 + CD45 + memory T lymphocytes	No change in CD4 + lymphocytes
	Increased CD4 + CD8 + cytotoxic T lymphocytes	No change in CD4 + lymphocytes
	Decreased CD19 + B lymphocytes	Increased plasma cells
	Increased eosinophils	Increased eosinophils
	No neutrophilic component	Increased neutrophils
	Increased mucosal mast cells	No change in mucosal mast cells
	Increased IELs	Increased IELs
	Increased EECs	Increased EECs (+/−)
	Increased ECs	Increased ECs (+/−)
Age of onset	<60 years (mature adult)	<5 years (juvenile)
Sex predilection	Female (+/−)	Male
Bacterial factors	Severity and duration of initial episode	Under investigation
	Antimicrobial administration	Under investigation
	CdtB	Under investigation
Associated genetic polymorphisms	TLR9	Unknown
	IL6	Unknown
	CDH1	Unknown
Increased intestinal permeability	Yes	Unknown
Treatment modalities	Symptom specific management: Antispasmodics	Empiric antimicrobial and anthelmintic therapy
	Symptom specific management: Anti-diarrheals	Symptom specific management: Anti-diarrheals; Fluid therapy
	Symptom specific management: Probiotics	Symptom specific management: Prebiotics and probiotics
	Symptom specific management: Serotonin receptor agonists	Experimental therapies: Fecal bacterial transfer; helminth exposure
	Psychological therapy	Experimental preventative therapy: *Campylobacter* spp. vaccination
	Dietary management/Food elimination trials	Dietary management
Clinical resolution	Eventual	Clinical progression to euthanasia

PI-IBS, post-infectious irritable bowel syndrome; CE, chronic enterocolitis; IEL, intraepithelial lymphocytes; EECs, enteroendocrine cells; ECs, enterochromaffin cells; CdtB, *Campylobacter* sp. cytolethal distending toxin subunit B. (+/−) indicates disagreement between published studies on the subject.

Another major knowledge gap is establishing *Campylobacter* sp. infection as a definitive instigating factor in the development of CE, as it has been in PI-IBS. Existing studies have suggested a link between *Campylobacter* and CE, with some studies reporting increased *Campylobacter* sp. prevalence in animals that went on to develop CE compared to healthy animals, increases in *Campylobacter* sp. in general in animals with CE, and specifically, more virulent *Campylobacter* strains circulating in animals with CE. However, it has been difficult to attribute causality to *Campylobacter* infection rather than it being simply a byproduct of dysbiosis associated with intestinal inflammation. Some studies suggest dysbiosis results in the loss of normal intestinal adherent bacteria, *H. macacae*, and replacement with *Campylobacter* sp. which could compound symptoms of chronic inflammation. In this scenario, *Campylobacter* infection is not necessarily the inciting cause of the syndrome. Human studies during large outbreaks have definitively linked *Campylobacter* infection to PI-IBS, and recent studies have even shown strain-level differences that may increase an individual’s risk of developing the syndrome. A recently published study from our group prospectively collected samples from rhesus macaques with and without CE to identify potential differences in *Campylobacter* species, bacterial quantities, and, using whole genome sequencing, any gene level strain differences between isolates collected from the two groups of animals. We identified *C. jejuni* and *C. coli* in animals both with and without intestinal disease, but quantities of both were increased in animals with intestinal disease. These increases were seen both in animals with CE, and in animals that went on to develop CE after sampling, supporting a potential role for *Campylobacter* in the development of disease. We also only found multilocus sequence type (MLST) 5,377 in animals with intestinal disease, and other isolates were only present in clinically healthy animals, suggesting the need for further investigation into strain specific factors as initiating components of CE, as has been suggested for PI-IBS (15, 115, 118, 119). Ultimately, if the histologic and *Campylobacter* characteristics are proven promising for a PI-IBS-like syndrome then complete characterization of the model will also require investigating other qualifying factors such as confirming visceral sensitivity, hypermotility, and host genetic factors involved in the development of the syndrome.

## Discussion

CE and PI-IBS are significant health concerns in rhesus macaque colonies and the human population, respectively. CE results in significant loss of breeding and research capacity in rhesus macaque colonies, and PI-IBS affects millions of people in the USA alone every year. Both represent multifactorial, difficult to diagnose and manage diseases with a distinct need for more investigation into their pathogeneses. The field of CE research would specifically benefit from investigations into heritability, further exploration into hormonal contributions, and confirmation or definitive evidence against infectious disease as an instigating factor. This would allow identification of biomarkers to identify individuals at high risk of developing the disease, and the development of novel management and treatment strategies. While existing animal models using various rodent species have succeeded in reproducing the host aspects of PI-IBS, particularly motility dysfunction, visceral hypersensitivity, and specific histopathologic characteristics, these models are chemically induced, which ignores the bacterial inciting factors for the development of the disease. If infectious disease can be proven as an initiating factor for CE, given the similarities in clinical syndromes, predilections for stress-related disease, and similarities in histologic findings between CE and PI-IBS, CE in rhesus macaques has the potential to act as a naturally occurring model for PI-IBS. With NHPs playing an active role in the pharmaceutical development pipeline, continued investigation and characterization of CE has the potential to vastly improve both animal and human health.
